# Ensino do Raciocínio Clínico Orientado pela Teoria dos Scripts de Doenças

**DOI:** 10.36660/abc.20220419

**Published:** 2022-11-09

**Authors:** Juliana de Cássia Vaz Oliveira, Aline Barbosa Peixoto, Gustavo Eugênio Martins Marinho, José Maria Peixoto

**Affiliations:** 1 Universidade José do Rosário Vellano Belo Horizonte MG Brasil Universidade José do Rosário Vellano , Campus Belo Horizonte, Belo Horizonte , MG – Brasil; 2 Universidade José do Rosário Vellano Alfenas MG Brasil Universidade José do Rosário Vellano – Alfenas, Alfenas , MG – Brasil

**Keywords:** Doenças Cardiovasculares, Educação Médica, Aprendizagem, Resolução de Problemas, Tomada de Decisão Clínica, Estudantes de Medicina

## Abstract

**Fundamento:**

O ensino do raciocínio clínico (RC) pode ser facilitado por estratégias educacionais orientadas pela teoria dos *scripts* de doenças (SD).

**Objetivo:**

Avaliar o efeito de uma estratégia educacional guiada pela teoria dos SD na acurácia diagnóstica (AD) da dor torácica (DT) em estudantes de medicina.

**Métodos:**

Estudo experimental em 3 fases, com 18 estudantes do 3º ano concluindo a fase 3, visto que as fases 1 e 2 tiveram 27 alunos. Na fase 1, cada participante resolveu 8 casos clínicos (6 de DT e 2 distratores). Na fase 2,os participantes foram divididos em 2 grupos, que treinaram distintamente 3 dos diagnósticos de DT da fase 1.Na fase 3, após uma semana, cada participante resolveu 8 novos casos, com os mesmos diagnósticos da fase 1.O tempo de resolução dos casos (TRC) e a AD foram avaliados. O nível de significância adotado na análise estatística foi p < 0,05.

**Resultados:**

Na fase 3, foram observadas melhora da AD e redução do TRC para os diagnósticos treinados em ambos os grupos, não ocorrendo transferência de aprendizagem. Para esses diagnósticos, os escores de AD nas fases 1 e 3 foram: grupo 1 = 1,00, IIQ [0,00-1,00] *versus* 2,00, IIQ [2,00-2,50]; p = 0,017 e grupo 2 = 1,00, IIQ [0,66-1,17] *versus* 3,00, IIQ [1,33-3,00]; p = 0,006. O TRC em segundos foram: Grupo 1: 485, IIQ [450-583] versus 318, IIQ [284-418]; p = 0,027 e grupo 2: 655, IIQ [543-740] *versus* 408, IIQ [337-569]; p = 0,010.

**Conclusão:**

A estratégia parece contribuir para melhora da AD e pode ser considerada para o ensino do RC.

## Introdução

O raciocínio clínico (RC) é um dos elementos determinantes da competência profissional. ^1^ Durante a graduação não é possível controlar a variabilidade de casos clínicos que os alunos defrontarão e os métodos de ensino do RC. ^2^ Acredita-se que os estudantes deveriam aprender a distinguir mais de 700 tipos de doenças. ^3^ O RC depende do nível de conhecimentos específicos organizados como *scripts* de doenças (SD) na memória de longo prazo. ^4^

Os SD constituem um sistema de conceitos que organizam os conhecimentos em relação a um diagnóstico. Frente a um caso clínico, os SD são ativados procurando-se relacioná-los ao caso atual. Para diagnósticos rotineiros, o processo ocorre automaticamente, com boa acurácia e pouco esforço cognitivo. Frente a doenças incomuns, haverá maior esforço mental, pois as informações serão avaliadas individualmente. A expertise diagnóstica se relaciona à variabilidade e qualidade dos SD adquiridos. ^1^

A formação dos SD ocorre em estágios. Inicialmente os estudantes aprendem os conhecimentos específicos das doenças. ^4^ Ao iniciarem as atividades assistenciais, passam a relacionar as manifestações clínicas ao conhecimento biomédico, que com a prática será “encapsulado” em padrões organizados como SD. ^4 - 7^ Estratégias para o desenvolvimento dos SD vêm sendo estudadas como: a reflexão estruturada (RE), a autoexplicação, os *scripts* de concordância, estudo de casos exemplos, dentre outras. ^5^ Estudos sobre a eficácia destas intervenções são limitados e ainda não há uma padronização para o ensino do RC. ^6 , 7^

Uma vez que estratégias guiadas pela teoria dos SD contribuem para o desenvolvimento do RC, ^8^ este estudo avaliou uma metodologia que procurou mimetizar os estágios do desenvolvimento dos SD. Foi testado ainda se o treinamento para doenças que comungam apresentações clínicas melhoraria a acurácia diagnóstica (AD) para doenças não treinadas com a mesma manifestação.

## Métodos

**Figura 1:**
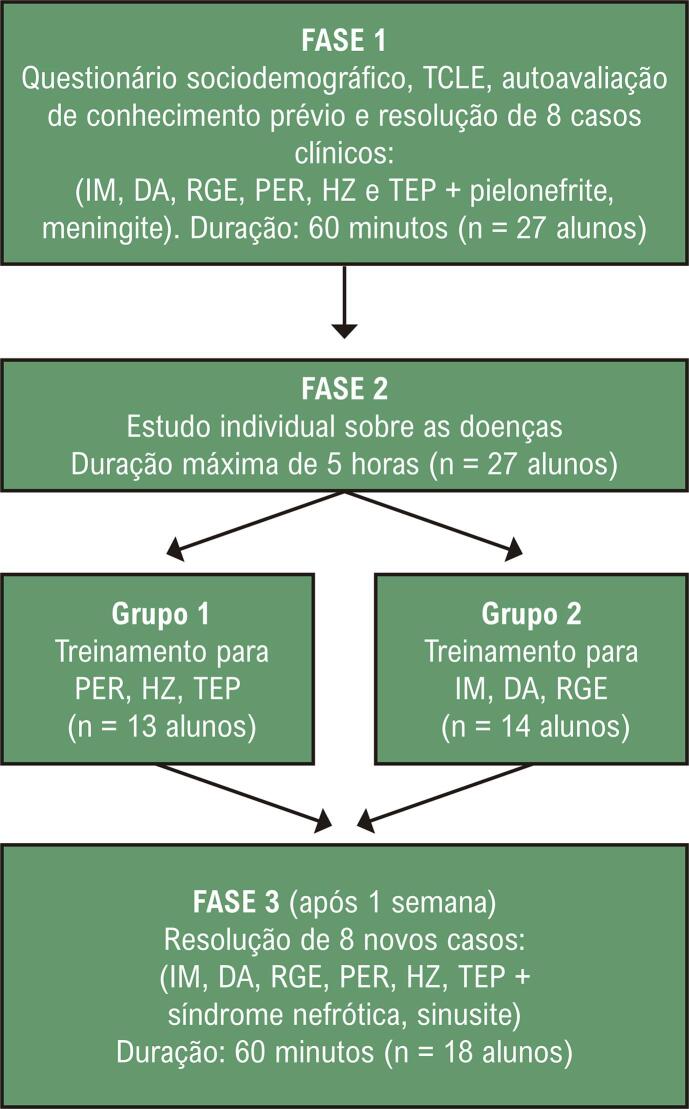
Desenho do estudo.

Estudo experimental com 3 fases ( Figura 1 ). Foram convidados alunos do 5º período de Medicina da UNIFENAS-BH (80 alunos), no 2° semestre de 2017, ao final do bloco de Pediatria, antes de iniciarem os blocos de Cardiologia, Pneumologia e Gastroenterologia. Estes estudantes foram escolhidos por ainda não terem sido expostos aos conhecimentos das doenças que fariam parte do estudo. Foram incluídos os que assinaram o Termo de Consentimento Livre e Esclarecido (TCLE), participaram em todas as fases do estudo e que não haviam cursado o 5º período. Foi assegurado-lhes o sigilo das informações.

Fonte: Elaborada pelos autores. Nota: TCLE: Termo de Consentimento Livre e Esclarecido; RGE: refluxo gastroesofágico; PER: pericardite; HZ: herpes-zóster; TEP: embolia pulmonar; IM: infarto do miocárdio; DA: dissecção aórtica.

### Metodologia instrucional

Com o objetivo de reproduzir os estágios de desenvolvimento dos SD, postulou-se que os estudantes deveriam inicialmente entrar em contato com os conhecimentos específicos das doenças que fariam parte do estudo: epidemiologia, fisiopatologia, manifestações clínicas e propedêutica. Posteriormente, através da RE, contrastariam suas características discriminatórias, ^2^ A seguir, praticariam exercícios de identificação, associação e categorização das doenças. Por fim, organizariam os conceitos estudados em mapas mentais (MM). ^9^

### Instrumentos

Foram utilizados 2 conjuntos de 8 casos clínicos, um para fase 1 e outro para a fase 3. Os casos foram apresentados em brochuras em sequência variável para evitar o vício de apresentação. O material continha instruções e um caso exemplo. Os casos abordavam 6 diagnósticos de dor torácica (DT) e continham aproximadamente 250 palavras informando a história clínica, exame físico e propedêutica para as seguintes doenças: infarto do miocárdio (IM), dissecção aórtica (DA), refluxo gastroesofágico (RGE), pericardite (PER), herpes-zóster (HZ) e embolia pulmonar (TEP). Os casos foram elaborados a partir de casos reais e validados por 3 especialistas. Foram inseridos 2 diagnósticos que não fizeram parte do estudo para reduzir o efeito de repetição da apresentação clínica (pielonefrite e meningite na fase 1; sinusite e síndrome nefrótica na fase 3).

### Procedimentos

#### Autoavaliação do conhecimento prévio dos participantes

Após assinar o TCLE e responder ao questionário sociodemográfico, os participantes realizaram a autoavaliação do conhecimento em relação às doenças do estudo através de uma escala de 5 pontos, na qual 1 = nunca estudei ou vi pacientes com esta doença e 5 = estudei ou vi frequentemente pacientes com esta doença. Nesse instrumento, as doenças do estudo estavam listadas em meio a outras, para evitar associação aos diagnósticos que seriam utilizados.

#### Fase 1 (avaliação inicial)

Nesta fase, após leitura de cada caso, os alunos forneceram 1 diagnóstico principal e 2 diferenciais de forma livre. Antes de iniciar a resolução de cada caso, foram instruídos a anotar os números que constavam em um cronômetro projetado à frente na sala e, ao término anotar novamente os números do cronômetro. Assim, foram aferidos os tempos de resolução dos casos (TRC).

#### Fase 2 (treinamento)

Os alunos foram divididos aleatoriamente em grupo 1 (G1) e grupo 2 (G2), selecionando-se sucessivamente o primeiro e último aluno da lista de presença. Os grupos foram alocados em salas separadas, onde o G1 treinou os diagnósticos de PER, TEP e HZ, e o G2 de IM, DA e RGE.

#### Estudo individual (duração: 60 minutos)

Inicialmente os alunos foram expostos aos componentes dos SD dos diagnósticos que seriam treinados (epidemiologia, fisiopatologia, manifestações clínicas e laboratoriais), através do estudo individual de uma apostila confeccionada pelos pesquisadores a partir de um livro de medicina interna. ^10^

#### Reflexão estruturada (duração: 60 minutos)

Após o estudo individual, os alunos compararam as doenças estudadas através da RE. Para isto, receberam um quadro onde deveriam identificar os fatores discriminatórios destas doenças, utilizando as informações da apostila que podia ser consultada. Os alunos foram instruídos a preencher o quadro no sentido horizontal favorecendo a comparação das doenças ( Quadro 1 ).

**Quadro 1:** Exercício de Reflexão Estruturada

Fatores definidores e Discriminatórios	Doença 1	Doença 2	Doença 3
Epidemiologia			
História clínica			
Exame físico			
Fisiopatologia			
Exames complementares			

Fonte: Elaborado pelos autores

#### Exercícios de identificação e associação (duração: 60 minutos)

Posteriormente, os estudantes receberam um material que apresentava aleatoriamente os elementos dos SD dos diagnósticos estudados. Foram orientados a sinalizar em um espaço reservado a qual(ais) diagnóstico(s) cada dado se relacionava. Propositalmente, foram inseridos dados que não pertenciam às doenças estudadas ( Quadro 2 ).

**Quadro 2:** Exercícios de identificação e associação referente à epidemiologia para as doenças: tromboembolismo, herpes-zóster e pericardite

Comum no paciente internado	[___, ___,___]		Doença pulmonar obstrutiva crônica	[___, ___,___]
Relação com envelhecimento	[___, ___,___]		Exposição ao sol	[___, ___,___]
Doença benigna	[___, ___,___]		Comum após cirurgia ortopédica	[___, ___,___]
Contato com águas de rio	[___, ___,___]		AIDS	[___, ___,___]
Hipertensão arterial	[___, ___,___]		Causa de grande morbidade	[___, ___,___]
Sofrimento por dor	[___, ___,___]		Anticoncepcional	[___, ___,___]
Doença autolimitada	[___, ___,___]		Viagens prolongadas	[___, ___,___]
Causa de morte na internação	[___, ___,___]		Não é comum a recorrência	[___, ___,___]
Comum em adultos jovens	[___, ___,___]		Obesidade	[___, ___,___]
Acidente vascular encefálico	[___, ___,___]		Pode ocorrer por doença não viral	[___, ___,___]
Unha encravada	[___, ___,___]		Complica pacientes com câncer	[___, ___,___]
Comum em pós-operatório	[___, ___,___]		Relação com infecção viral	[___, ___,___]

Fonte: Elaborado pelos autores. Nota: Os estudantes foram orientados a escrever à frente de cada dado, a(s) letra(s) correspondente(s) à doença com as quais se relacionam. Neste caso: T: tromboembolismo; H: herpes-zóster; P: pericardite.

#### Mapas Mentais (duração: 60 minutos)

**Figura 2:**
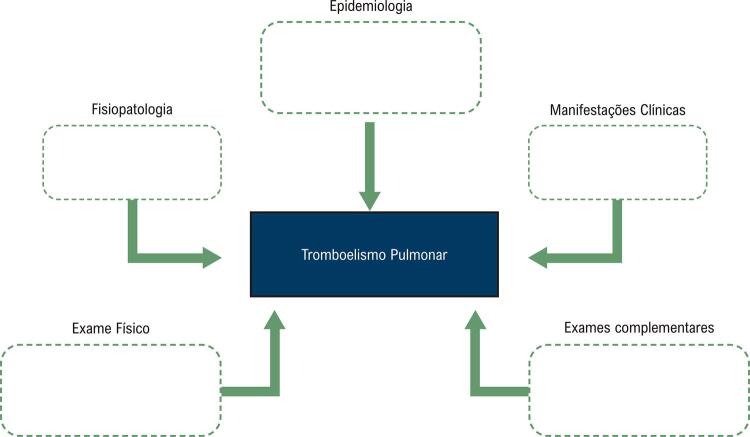
Mapa mental para o diagnóstico do tromboembolismo pulmonar.

Nesta fase os estudantes construíram os MM das doenças treinadas. No centro do mapa foi colocado o diagnóstico, e partindo deste, foram desenvolvidos ramos correspondentes aos elementos dos SD. Em cada ramo, havia uma área onde os alunos deveriam descrever as características relacionadas ao diagnóstico ( Figura 2 ).

Fonte: Elaborada pelos autores.

#### Aplicação na resolução de casos clínicos (duração: 60 minutos)

Por fim, os estudantes revisaram as vinhetas da fase 1, fornecendo novamente os diagnósticos principais e diferenciais. Os materiais de estudo podiam ser consultados.

#### Fase 3 (avaliação tardia)

Após 1 semana os participantes resolveram 8 novos casos, com os diagnósticos da fase 1 e 2 novos distratores. Após a leitura de cada vinheta forneceram 1 diagnóstico principal e 2 diferenciais. O TRC foi aferido pelo mesmo procedimento da fase 1.

## Aspectos éticos

Este trabalho foi aprovado pelo Comitê de Ética em Pesquisa da UNIFENAS, com parecer número: 1.877.200 (CAAE: 60865316.8.0000.5143).

## Análise de dados

### Pontuação das respostas

Para mensurar a AD, os diagnósticos principais fornecidos nas fases 1 e 3 foram listados e pontuados independentemente por 3 clínicos. Foi utilizado um sistema de 3 pontos no qual: 1 ponto foi atribuído se o diagnóstico estivesse correto; 0,5 se o diagnóstico não fosse citado, mas um constituinte fosse mencionado (ex. isquemia em um caso de IM) e 0 para o diagnóstico errado.

### Análise estatística

Para cada participante, a média das pontuações em cada diagnóstico foi computada obtendo-se 2 variáveis: a AD nas fases 1 e 3. Como os grupos trabalharam diagnósticos diferentes, procedeu-se a análise por blocos de doenças: bloco 1 (HZ, TEP, PER); bloco 2 (IM, RGE, DA); bloco 3 (HZ, TEP, PER, IM, RGE, DA). As variáveis categóricas são apresentadas em números e percentuais. As variáveis contínuas sem distribuição normal são apresentadas como mediana e intervalo interquartil (IIQ). Para verificação da normalidade dos dados, utilizou-se o Teste de Shapiro-Wilk. Uma vez que a normalidade dos dados não foi confirmada, os testes não-paramétricos de Mann-Whitney para amostras independentes e de Wilcoxon para amostras pareadas foram utilizados. A comparação dos participantes quanto à idade e à autoavaliação de conhecimento prévio foi realizada pelo teste de Mann-Whitney; quanto ao sexo, pelo teste exato de Fisher. Para avaliar o efeito da intervenção em cada grupo foi utilizado o teste de Mann-Whitney. A eficácia da estratégia proposta nas pontuações entre as fases foi avaliada pelo teste de Wilcoxon. Os resultados foram considerados significativos para uma probabilidade de significância < 5%. A análise estatística foi realizada utilizando o software SPSS, versão 17.0.

## Resultados

### Caracterização sociodemográfica

Inicialmente, 27 alunos foram voluntários no estudo. Na fase 2, 13 alunos foram alocados no G1 e 14 no G2. Para a fase 3, retornaram 18 alunos, que constituíram o grupo considerado para análise dos dados, 7 do G1 e 11 do G2. A mediana da idade em anos era semelhante entre os grupos: G1 = 21, IIQ [20-26] *versus* G2 = 21, IIQ [20-60]; p = 0,96. O G1 incluiu 5 mulheres (71,4%) e o G2, 6 (60%); p = 1,00. As medianas da autoavaliação do conhecimento prévio não diferiram entre grupos: G1 = 2,67, IIQ [1,83-3,00] e G2 = 3,00, IIQ [2,50-3,67]; p = 0,24.

#### Acurácia diagnóstica na fase 1

Não havia diferença entre as medianas das pontuações obtidas entre os grupos em relação a cada um dos blocos de doenças na fase 1 ( Tabela 1 ).

**Tabela 1:** Análise comparativa da acurácia diagnóstica entre os grupos 1 e 2, por fase e bloco de doenças

Blocos de doenças e fase	Grupo 1 (n=7)	Grupo 2 (n=11)	p

Fase 1	Acurácia diagnóstica	Acurácia diagnóstica	
**Bloco 1** HZ, TEP, PER	1,00 [0,00-1,00]	1,00 [0,00-1,00]	0,961
**Bloco 2** IM, RGE, DA	1,00 [0,33-1,83]	1,00 [0,66-1,17]	0,747
**Bloco 3** HZ, TEP, PER, IM, RGE, DA	1,83 [1,00-2,00]	1,66 [1,00-3,00]	0,819
**Fase 3**	**Acurácia diagnóstica**	**Acurácia diagnóstica**	
**Bloco 1** HZ, TEP, PER	2,00 [2,00-2,50]	1,00 [0,00-1,00]	0,004
**Bloco 2** IM, RGE, DA	1,00 [1,00-2,83]	3,00 [1,33-3,00]	0,041
**Bloco 3** HZ, TEP, PER, IM, RGE, DA	3,00 [2,00-4,83]	4,00 [1,33-4,00]	0,791

Fonte: dados do estudo. Nota: Base de dados: 18 alunos; p: teste de Mann-Whitney; Variáveis numéricas: mediana [intervalo interquartil]; n: número de alunos; HZ: herpes-zóster; TEP: embolia pulmonar; PER: pericardite; IM: infarto do miocárdio; RGE: refluxo gastroesofágico; DA: dissecação aórtica; variação da pontuação: bloco 1 e 2 (0 a 3); bloco 3 (0 a 6).

#### Acurácia diagnóstica na fase 3

A Tabela 1 demonstra que foram observadas diferenças entre grupos quanto à pontuação da AD obtida na fase 3. O G1, que treinou as doenças do bloco 1, obteve pontuação maior para estes casos na fase 3, comparado ao G2. O inverso ocorreu no G2 que treinou as doenças do bloco 2. No bloco 3 não houve diferença entre grupos.

Analisada a AD entre as fases 1 e 3 por grupo e bloco de doenças ( Tabela 2 ), observa-se no G1 que a mediana para os diagnósticos do bloco 1 é maior na fase 3, sem diferença para o bloco 2. No G2, a mediana para os diagnósticos do bloco 2 é maior na fase 3, sem diferença para o bloco 1.

**Tabela 2:** Análise comparativa da acurácia diagnóstica entre as fases 1 e 3, por grupo e bloco de doenças

Blocos de doenças por Grupo	Fase 1	Fase 3	p

Fase 1	Acurácia diagnóstica	Acurácia diagnóstica	
**Grupo 1 (n=7)**			
**Bloco 1** HZ, TEP, PER	1,00 [0,00-1,00]	2,00 [2,00-2,50]	0,017
**Bloco 2** IM, RGE, DA	1,00 [0,33-1,83]	1,00 [1,00-2,83]	0,450
**Bloco 3** HZ, TEP, PER, IM, RGE, DA	1,83 [1,00-2,00]	3,00 [2,00-4,83]	0,027
**Grupo 2 (n=11)**	**Acurácia diagnóstica**	**Acurácia diagnóstica**	
**Bloco 1** HZ, TEP, PER	1,00 [0,00-1,00]	1,00 [0,00-1,00]	0,854
**Bloco 2** IM, RGE, DA	1,00 [0,66-1,17]	3,00 [1,33-3,00]	0,006
**Bloco 3** HZ, TEP, PER, IM, RGE, DA	1,66 [1,00-3,00]	4,00 [1,33-4,00]	0,006

Fonte: dados do estudo. Base de dados: 18 alunos; p: teste de Wilcoxon; variáveis numéricas: mediana [intervalo interquartil]; n: número de alunos; HZ: herpes-zóster; TEP: embolia pulmonar; PER: pericardite; IM: infarto do miocárdio; RGE: refluxo gastroesofágico; DA: dissecção aórtica; variação da pontuação: bloco 1 e 2 (0 a 3); bloco 3 (0 a 6).

#### Tempo de resolução dos casos

A Tabela 3 demonstra que, no G1, que treinou os casos do bloco 1, houve redução do TRC de todos os blocos na fase 3. Já no G2, que treinou os casos do bloco 2, ocorreu redução do TRC dos blocos 2 e 3 na fase 3.

**Tabela 3:** Tempo gasto na resolução dos casos entre as fases do estudo, por grupo e bloco de doenças

Blocos de doenças por Grupo	Fase 1	Fase 3	p

Grupo 1 (n=6)	Tempo em segundos	Tempo em segundos	
**Bloco 1** HZ, TEP, PER	485 [450 - 583]	318 [284-418]	0,027
**Bloco 2** IM, RGE, DA	558 [400 -1.067]	495 [181-646]	0,046
**Bloco 3** HZ, TEP, PER, IM, RGE, DA	1.059 [874 -1.744]	812 [466-1.064]	0,028
**Grupo 2 (n=11)**	**Tempo em segundos**	**Tempo em segundos**	
**Bloco 1** HZ, TEP, PER	501 [485-588]	421 [290-576]	0,328
**Bloco 2** IM, RGE, DA	655 [543-740]	408 [337-569]	0,010
**Bloco 3** HZ, TEP, PER, IM, RGE, DA	1.131 [1.020-1.317]	872 [698-1.062]	0,026

Fonte: dados do estudo. Base de dados: 17 alunos (1 caso sem informação); p: teste de Wilcoxon; variáveis numéricas: mediana em segundos [intervalo interquartil]; n: número de alunos; HZ: herpes-zóster; TEP: embolia pulmonar; PER: pericardite; IM: infarto do miocárdio; RGE: refluxo gastroesofágico; DA: dissecção aórtica.

## Discussão

Este estudo avaliou o efeito de uma abordagem instrucional orientada pela teoria dos SD na AD para casos de DT em estudantes de medicina. Os resultados confirmaram que a metodologia melhorou a AD dos estudantes e diminuiu o TRC, sugerindo aquisição da representação mental (RM) para as doenças treinadas, em conformidade com a teoria dos SD. ^11^ No entanto, a transferência da aprendizagem (TA) a um grupo de doenças não treinadas com a mesma apresentação clínica não foi confirmada.

Outros estudos se orientaram pela teoria dos SD, no entanto este é um dos primeiros a mimetizar os estágios do seu desenvolvimento. Moghadami et al., ^12^ compararam o ensino do RC, orientado pelos SD ao ensino tradicional em estudantes do 4º ano. O grupo intervenção, após a leitura de um caso clínico, foi orientado a identificar a RM do problema e comparar os componentes dos SD para 3 diagnósticos diferenciais, enquanto o grupo controle assistiu uma aula expositiva sobre as doenças do estudo e discutiram em pequenos grupos. A atividade durou 7 horas e foi constatado que ambos os grupos melhoraram a AD, mas o grupo intervenção superou o controle.

Em nosso estudo, os participantes eram menos experientes, estavam no início do 3º ano e não haviam iniciado o ciclo clínico. Provavelmente para estes alunos seria difícil identificar a RM de um problema, por exigir habilidade de inferência e, portanto, maior conhecimento sobre as doenças. Talvez em uma fase pré-clínica, uma metodologia que orientasse as operações cognitivas para a elaboração dos SD fosse mais adequada e uma guiada para a RM dos problemas poderia ser implementada nos anos subsequentes. Estas questões poderão ser avaliadas em estudos futuros.

Em outro estudo orientado pela teoria dos SD, ^13^ 15 estudantes de medicina do 4° ano e 12 do 6º ano participaram de uma aula sobre a teoria dos SD. Posteriormente, os alunos do 4º ano, após a leitura de casos clínicos que comungavam diagnósticos diferenciais, informaram as características clínicas (CC) comuns e discriminatórias de cada caso. Aos estudantes do 6º ano foi solicitado informar 2 diagnósticos, as CC dos diagnósticos e o grau de predição das CC informadas. Os estudantes receberam *feedback* durante a atividade, que durou 3 horas.

Os resultados demonstraram que houve melhora da habilidade dos alunos do 6º ano para identificar novas CC das doenças, sem melhora na AD e do reconhecimento das CC discriminatórias. Entre os estudantes do 4º ano, a atividade não demonstrou benefício. ^13^ Diferente do nosso estudo, a atividade foi direcionada para a identificação das CC das doenças, com melhora na AD nos alunos mais avançados. Talvez para estudantes menos experientes, uma metodologia que forneça mais suporte, como a desenvolvida em nosso trabalho, teria mais impacto. Estas considerações poderão ser avaliadas em futuros estudos.

Outros estudos não guiados pela teoria dos SD obtiveram resultados satisfatórios, como o realizado por Diemers et al., ^14^ que desenvolveram um curso de RC com duração de 10 semanas. Neste, os estudantes explicavam em voz alta a fisiopatologia, enquanto analisavam 4 casos (2 do curso e 2 de transferência). Semelhante aos nossos achados, foi observada melhoria na AD dos alunos, com redução do TRC, mas a aprendizagem não foi transferida a casos não treinados. Uma vantagem da estratégia proposta no presente estudo é a duração máxima de 5 horas, exequível em ambientes educacionais.

Keemink et al., ^15^ investigaram a TA de RC em um curso baseado em casos clínicos. Após explicarem em voz alta a fisiopatologia, os fatores predisponentes, as CC, a propedêutica e o manejo de 15 doenças (5 do curso), os estudantes analisaram 12 vinhetas clínicas, 4 com diagnósticos treinados no curso. Semelhante aos nossos dados, houve melhora da AD apenas para as doenças treinadas. O debate sobre a TA de um contexto a outro não é novo. ^16^ Por TA entende-se o uso das habilidades aprendidas a uma nova situação, que necessita da recontextualização do conhecimento, um dos últimos estágios do aprendizado. ^17^

Como mencionando, o RC ocorre pelo reconhecimento dos SD, que contêm as CC discriminatórias das doenças. ^4^ Estudos sobre transferência analógica sugerem que as CC possuem elementos superficiais e profundos. Os elementos profundos se relacionam às regras que determinam um diagnóstico e os superficiais às manifestações clínicas. Para a TA é necessário a identificação dos elementos profundos, entretanto, são os superficiais os mais perceptíveis. ^17^ Este fato pode ter impedido, em nosso estudo, a TA para as doenças não treinadas, pois apesar de comungarem as manifestações clínicas, os diagnósticos possuíam CC distintas em relação à epidemiologia, fisiopatologia e propedêutica. Portanto, o aluno não foi capaz de chegar a um diagnóstico correto, pois não tinha disponível os conhecimentos específicos (profundos) destas doenças. Em medicina não existe uma habilidade geral para a solução de todas as tarefas, pois possuem conteúdos específicos. ^17^

Neste estudo, para reproduzir os estágios do desenvolvimento dos SD, foram utilizadas estratégias educacionais sugeridas para o ensino do RC. ^5^ A RE baseia-se na aprendizagem experiencial que envolve: percepção, descrição, análise e síntese. Requer a intencionalidade do estudante na procura de evidências que apoiem seu aprendizado. ^18^ No ensino do RC, a RE favorece a comparação das características das doenças contribuindo para a aquisição da RM destas. ^19^ Outra estratégia utilizada foi o treino cognitivo, que tinha por objetivo o exercício de algumas habilidades do pensamento, ^20 , 21^ como a atenção, percepção, codificação, memória, raciocínio e criatividade. Resultados favoráveis como esta metodologia já foram relatados para habilidades cirúrgicas. ^20 , 21^ Este é um dos primeiros estudos a utilizar esta metodologia no ensino do RC, área que merece novas investigações.

Ao final, os estudantes construíram MM dos diagnósticos treinados, que facilitam a visualização de como as informações se relacionam, melhorando a memorização do conteúdo. ^9^ Kalyanasundaram et al., ^22^ demonstraram que MM melhoram a recordação das informações uma semana após uma atividade instrucional. Os MM foram pouco testados no ensino do RC, mas acreditamos que possa ter favorecido a visualização da RM das doenças, uma vez que sua construção considerou os componentes dos SD.

Esse estudo ressalta a importância de uma atividade estruturada para o desenvolvimento do RC. Os resultados são encorajadores, já que a literatura enfatiza a necessidade do contato real com pacientes para aquisição dos SD. ^15^ Nossos resultados demonstraram que, mesmo antes de iniciarem as atividades clínicas, os estudantes beneficiam-se de um programa de RC, que pode servir como ponte para o início do ciclo clínico.

A metodologia proposta demonstrou resultados satisfatórios para o ensino do RC e permitiu o exercício de diagnósticos variados e a manipulação dos componentes dos SD, fato relevante para a aquisição de uma rede de SD. ^1 , 4^ A atividade apresenta duração adequada para ambientes educacionais e sua incorporação a plataformas computacionais contribuiria para maior interação e *feedback* . Iniciativas neste sentido vem sendo desenvolvidas como a Clinical Key, ^23^ NEJM Healer ^24^ e Paciente 360. ^25^

O presente trabalho possui limitações. Trata-se de estudo único neste formato, com pequeno número de participantes ao final. Como foi planejado iniciá-lo antes dos blocos de especialidades, as atividades concorreram com as provas finais de Pediatria que antecedem estes blocos, fato que contribuiu para perda amostral. Desse modo, sua replicação com maior número de participantes contribuiria para a confirmação dos resultados. O método foi testado para o diagnóstico da DT, sendo necessário avaliar seu uso em outras condições.

Não é possível identificar a contribuição de cada estratégia empregada isoladamente. Pode-se argumentar que a melhoria do desempenho se deve a um efeito geral do esforço investido na atividade, em vez de um resultado específico atribuído à metodologia. Embora os estudantes tenham dedicado esforço à atividade, o que importa é que houve aquisição da habilidade para a qual foram treinados e que o formato pode ser mais atraente em relação a métodos tradicionais comumente utilizados. Trabalhar com casos clínicos é representativo da prática futura dos alunos e pode favorecer o desenvolvimento do RC. Estimulante foi o *feedback* recebido pelos estudantes solicitando novas sessões para outros diagnósticos.

## Conclusão

A abordagem instrucional proposta melhorou a AD dos estudantes para o diagnóstico da DT. No entanto, a melhoria ocorreu apenas para as doenças treinadas, não sendo observado a TA. A estratégia é de fácil execução e pode ser considerada para o desenvolvimento do RC.
