# Supplementary material for: Ensino do Raciocínio Clínico Orientado pela Teoria dos Scripts de Doenças
Source: Arq Bras Cardiol. 2022 Nov 9;119(5 Suppl 1):14–21. [Article in Portuguese] doi: 10.36660/abc.20220419 (PMC9750199; doi:10.36660/abc.20220419)
Supplement: Translation

# Translation: Teaching of Clinical Reasoning Guided by Illness Script Theory

**Authors:** Juliana de Cássia Vaz Oliveira, Aline Barbosa Peixoto, Gustavo Eugênio Martins Marinho, José Maria Peixoto

**Affiliations:** 1 Brazil Universidade José do Rosário Vellano, Campus Belo Horizonte, Belo Horizonte, MG – Brazil; 2 Brazil Universidade José do Rosário Vellano – Alfenas, Alfenas, MG – Brazil

**Keywords:** Cardiovascular Diseases, Education,Medical, Learning, Decision Making, Clinical Decision Making, Students, Medical

## Abstract

**Background:**

Teaching of clinical reasoning (CR) can be facilitated by educational strategies guided by illness script theory.

**Objective:**

To evaluate the effects of an educational strategy guided by illness script theory on the diagnostic accuracy of chest pain in medical students.

**Methods:**

Experimental study in 3 phases, with 18 third-year medical students completing phase 3. Phases 1 and 2 had 27 students. In phase 1, each participant solved 8 clinical cases (6 of chest pain and 2 distractors). In phase 2, participants were divided into 2 groups, which distinctly trained 3 of the chest pain diagnoses from phase 1. In phase 3, after 1 week, each participant solved 8 new cases, with the same diagnoses as phase 1. Case resolution time and diagnostic accuracy were evaluated. The significance level adopted for statistical analysis was p < 0.05.

**Results:**

In phase 3, both groups showed improved diagnostic accuracy and reduced case resolution time for the trained diagnoses, with no transfer of learning. For these diagnoses, the diagnostic accuracy scores in phases 1 and 3 were: group 1 = 1.00, IQR [0.00 to 1.00] versus 2.00, IQR [2.00 to 2.50], p = 0.017 and group 2 = 1.00, IQR [0.66 to 1.17] versus 3.00, IQR [1.33 to 3.00], p = 0.006. Case resolution times in seconds were: group 1: 485, IQR [450 to 583] versus 318, IQR [284 to 418], p = 0.027 and group 2: 655, IQR [543 to 740] versus 408, IQR [337 to 569], p = 0.010.

**Conclusion:**

The proposed strategy seems to contribute to improved diagnostic accuracy, and it may be considered for teaching CR.

## Introduction

Clinical reasoning (CR) is a determining element of professional competence.^1^ During the undergraduate course, it is not possible to control the variability of clinical cases that students will face or the teaching methods of CR.^2^ It is believed that students should learn to distinguish more than 700 types of illnesses.^3^ CR depends on the level of specific knowledge organized as illness scripts in long-term memory.^4^

Illness scripts are a system of concepts that organize knowledge in relation to a diagnosis. Faced with a clinical case, illness scripts are activated in an attempt to relate them to the current case. For routine diagnoses, the process occurs automatically, with good accuracy and little cognitive effort. Faced with uncommon diseases, there will be greater mental effort, as the information will be evaluated individually. Diagnostic expertise is related to the variability and quality of illness scripts acquired.^1^

The formation of illness scripts occurs in stages. Initially, students learn specific knowledge about illnesses.^4^ When initiating care activities, they begin to relate clinical manifestations to biomedical knowledge, which will with practice be “encapsulated” in patterns organized as illness scripts.^4 - 7^ Strategies for the development of illness scripts have been studied, such as structured reflection, self-explanation, agreement scripts, study of example cases, and others.^5^ Studies on the effectiveness of these interventions are limited, and there is still no standardization for teaching CR.^6 , 7^

Given that strategies guided by illness script theory contribute to the development of CR,^8^ this study evaluated a methodology that sought to imitate the stages of illness script development. The study also tested whether training for illnesses that share clinical presentations would improve diagnostic accuracy for illnesses with the same manifestation that were not trained.

## Methods

This was an experimental study with 3 phases ( Figure 1 ). Students from the fifth period of Medicine at the UNIFENAS University in Belo Horizonte, Minas Gerais, Brazil (80 students) were invited in the second semester of 2017, at the end of the Pediatrics unit, before starting the Cardiology, Pulmonology, and Gastroenterology units. These students were chosen because they had not yet been exposed to knowledge of the illnesses that would be part of the study. The study included students who signed the free and informed consent form, who participated in all phases of the study, and who had not completed the fifth period. They were guaranteed confidentiality of information.

**Figure 1:** Study design.

Source: Designed by the authors. Note: AD: aortic dissection; FICF: free and informed consent form; GER: gastroesophageal reflux; HZ: herpes zoster; MI: myocardial infarction; PER: pericarditis; PTE: pulmonary thromboembolism.

### Instructional methodology

With the objective of reproducing the stages of development of illness scripts, it was postulated that students should initially be exposed to the specific knowledge of the illnesses that would be part of the study: epidemiology, pathophysiology, clinical manifestations, and clinical workup. Subsequently, through structured reflection, they would contrast their discriminatory characteristics.^2^ Afterwards, they would practice exercises to identify, associate, and categorize the illnesses. Finally, they would organize the concepts studied into mind maps.^9^

### Instruments

The study used 2 sets of 8 clinical cases, one for phase 1 and another for phase 3. The cases were presented in brochures in a variable sequence to avoid presentation bias. The material contained instructions and an example case. The cases addressed 6 diagnoses of chest pain, containing approximately 250 words that informed the clinical history, physical examination, and clinical workup for the following diseases: myocardial infarction (MI), aortic dissection (AD), gastroesophageal reflux (GER), pericarditis (PER), herpes zoster (HZ), and pulmonary thromboembolism (PTE). The cases were elaborated based on real cases, and they were validated by 3 experts. Two diagnoses that were not part of the study were inserted to reduce the effect of recurrence of clinical presentation (pyelonephritis and meningitis in phase 1; sinusitis and nephrotic syndrome in phase 3).

### Procedures

#### Participants’ self-assessment of prior knowledge

After signing the free and informed consent form and answering the sociodemographic questionnaire, participants completed a self-assessment of their knowledge of the illnesses in the study using a 5-point scale, in which 1 = I have never studied or seen patients with this illness and 5 = I have studied or frequently seen patients with this illness. In this instrument, the illnesses in the study were listed among others, to avoid association with the diagnoses that would be used.

#### Phase 1 (initial assessment)

In this phase, after reading each case, the students freely provided 1 main diagnosis and 2 differential diagnoses. Before beginning to solve each case, they were instructed to write down the numbers that appeared on a stopwatch projected at the front of the room and, at the end, write down the numbers on the stopwatch again. In this manner, case resolution times (CRT) were measured.

#### Phase 2 (training)

Students were randomly divided into group 1 (G1) and group 2 (G2), sequentially selecting the first and last student from the attendance list. The groups were allocated in separate rooms, where G1 trained diagnoses of PER, PTE, and HZ, and G2 trained MI, AD, and GER.

#### Individual study (duration: 60 minutes)

Initially, students were exposed to the components of the illness scripts for the diagnoses that would be trained (epidemiology, pathophysiology, and clinical and laboratory manifestations), through individual study of a handout prepared by the researchers based on an internal medicine book.^10^

#### Structured reflection (duration: 60 minutes)

Following individual study, the students compared the studied illnesses using structured reflection. To do this, they received a chart where they had to identify the discriminatory factors of these illnesses, using the information from the handout, which could be consulted. The students were instructed to fill in the table horizontally, favoring comparison of the illnesses ( Chart 1 ).

**Chart 1:** Structured reflection exercise

Defining and discriminatory factors	Illness 1	Illness 2	Illness 3
Epidemiology			
Clinical history			
Physical examination			
Physiopathology			
Complementary exams			

Source: Designed by the authors.

#### Identification and association exercises (duration: 60 minutes)

Subsequently, the students received material that randomly presented the elements of the illness scripts of the diagnoses studied. They were instructed to indicate, in a reserved space, to which diagnosis(es) each piece of data was related. Intentionally, data that did not belong to the illnesses studied were included ( Chart 2 ).

**Chart 2:** Identification and association exercises referring to the epidemiology for the illnesses thromboembolism, herpes zoster, and pericarditis

Common in hospitalized patients	[___, ___,___]	Chronic obstructive pulmonary disease	[___, ___,___]
Related to aging	[___, ___,___]	Exposure to the sun	[___, ___,___]
Benign illness	[___, ___,___]	Common after orthopedic surgery	[___, ___,___]
Contact with water from a river	[___, ___,___]	AIDS	[___, ___,___]
Arterial hypertension	[___, ___,___]	Cause of great morbidity	[___, ___,___]
Suffering from pain	[___, ___,___]	Contraceptive use	[___, ___,___]
Self-limited disease	[___, ___,___]	Extended travel	[___, ___,___]
Cause of death on admission	[___, ___,___]	Recurrence is not common	[___, ___,___]
Common in young adults	[___, ___,___]	Obesity	[___, ___,___]
Stroke	[___, ___,___]	May occur do to non-viral illness	[___, ___,___]
Ingrown nail	[___, ___,___]	Complication in patients with cancer	[___, ___,___]
Common during the postoperative period	[___, ___,___]	Related to viral infection	[___, ___,___]

Source: Designed by the authors. Note: Students were instructed to write, in front of each piece of data, the letter(s) corresponding to the illness(es) to which they relate. In this case: H: herpes zoster; P: pericarditis; T: thromboembolism.

#### Mind maps (duration: 60 minutes)

In this phase, students constructed mind maps for the trained illnesses. In the center of the map, the diagnosis was placed, and, from that point, branches corresponding to the elements of the illness scripts were developed. In each branch, there was an area where students should describe the characteristics related to the diagnosis ( Figure 2 ).

**Figure 2:** Mind map for diagnosis of pulmonary thromboembolism.

Source: Designed by the authors.

#### Application to the resolution of clinical cases (duration: 60 minutes)

Finally, the students reviewed the vignettes from phase 1, again providing the main and differential diagnoses. The study materials could be consulted.

#### Phase 3 (late assessment)

After 1 week, participants resolved 8 new cases, with the diagnoses from phase 1 and 2 new distractors. After reading each vignette, they provided 1 main diagnosis and 2 differential diagnoses. The CRT was measured using the same procedure as phase 1.

## Ethical aspects

This study received approval from the Research Ethics Committee of UNIFENAS, under opinion number: 1.877.200 (CAAE: 60865316.8.0000.5143).

## Data analysis

### Scoring of the answers

To measure diagnostic accuracy, the main diagnoses provided in phases 1 and 3 were listed and scored independently by 3 clinicians. A 3-point system assigned scores as follows: 1 point for correct diagnosis; 0.5 points if the diagnosis was not listed, but a component was mentioned (e.g., ischemia in a case of MI); and 0 points for incorrect diagnosis.

## Statistical analysis

For each participant, the average score for each diagnosis was calculated, obtaining 2 variables: diagnostic accuracy in phases 1 and 3. As the groups worked on different diagnoses, analysis was carried out by blocks of illnesses: block 1 (HZ, PTE, and PER), block 2 (MI, GER, and AD), and block 3 (HZ, PTE, PER, MI, GER, and AD). Categorical variables are presented as numbers and percentages. Continuous variables without normal distribution are presented as median and interquartile range (IQR). To verify the normality of the data, the Shapiro-Wilk test was used. Since the normality of the data was not confirmed, the non-parametric Mann-Whitney tests for independent samples and the Wilcoxon tests for paired samples were used. Comparison of participants in terms of age and self-assessment of prior knowledge was performed using the Mann-Whitney test; comparison in terms of sex used Fisher’s exact test. To assess the effect of the intervention in each group, the Mann-Whitney test was used. The effectiveness of the proposed strategy in relation to the scores between the phases was evaluated using the Wilcoxon test. Results with probability of significance < 5% were considered significant. Statistical analysis was performed using SPSS software, version 17.0.

## Results

### Sociodemographic characteristics

Initially, 27 students volunteered in the study. In phase 2, 13 students were allocated to G1 and 14 to G2. For phase 3, 18 students returned, constituting the group considered for data analysis, with 7 students from G1 and 11 from G2. The median age in years was similar between the groups: G1 = 21, IQR [20 to 26] versus G2 = 21, IQR [20 to 60]; p = 0.96. G1 included 5 women (71.4%), and G2 included 6 (60%); p = 1.00. The medians for self-assessment of prior knowledge did not differ between groups: G1 = 2.67, IQR [1.83 to 3.00] and G2 = 3.00, IQR [2.50 to 3.67]; p = 0.24.

### Diagnostic accuracy in phase 1

There was no difference between the median scores obtained between the groups in relation to each of the blocks of illnesses in phase 1 ( Table 1 ).

**Table 1:** Comparative analysis of diagnostic accuracy between groups 1 and 2, by phase and block of illnesses

Blocks of illnesses and phase	Group 1 (n=7)	Group 2 (n=11)	p

Phase 1	Diagnostic accuracy	Diagnostic accuracy	
**Block 1** HZ, PTE, PER	1.00 [0.00-1.00]	1.00 [0.00-1.00]	0.961
**Block 2** MI, GER, AD	1.00 [0.33-1.83]	1.00 [0.66-1.17]	0.747
**Block 3** HZ, PTE, PER, MI, GER, AD	1.83 [1.00-2.00]	1.66 [1.00-3.00]	0.819
**Phase 3**	**Diagnostic accuracy**	**Diagnostic accuracy**	
**Block 1** HZ, PTE, PER	2.00 [2.00-2.50]	1.00 [0.00-1.00]	0.004
**Block 2** MI, GER, AD	1.00 [1.00-2.83]	3.00 [1.33-3.00]	0.041
**Block 3** HZ, PTE, PER, MI, GER, AD	3.00 [2.00-4.83]	4.00 [1.33-4.00]	0.791

Source: Study data. Note: Database: 18 students; p: Mann-Whitney test; numerical variables: median [interquartile range]; AD: aortic dissection; GER: gastroesophageal reflux; HZ: herpes zoster; MI: myocardial infarction; n: number of students; PER: pericarditis; PTE: pulmonary thromboembolism; score variation: blocks 1 and 2 (0 to 3); block 3 (0 to 6).

### Diagnostic accuracy in phase 3

Table 1 shows that differences were observed between groups regarding the diagnostic accuracy score obtained in phase 3. G1, which trained the illnesses in block 1, obtained a higher score for these cases in phase 3, compared to G2. The opposite occurred in G2, which trained the diseases in block 2. For block 3, there was no difference between groups.

When analyzing the diagnostic accuracy between phases 1 and 3 by group and block of illnesses ( Table 2 ), in G1, the median for diagnoses in block 1 was higher in phase 3, with no difference in block 2. In G2, the median for diagnoses in block 2 was higher in phase 3, with no difference in block 1.

**Table 2:** Comparative analysis of diagnostic accuracy between phases 1 and 3, by group and block of illnesses

Blocks of illnesses by group	Phase 1	Phase 3	p

Phase 1	Diagnostic accuracy	Diagnostic accuracy	
**Group 1 (n=7)**			
**Block 1** HZ, PTE, PER	1.00 [0.00-1.00]	2.00 [2.00-2.50]	0.017
**Block 2** MI, GER, AD	1.00 [0.33-1.83]	1.00 [1.00-2.83]	0.450
**Block 3** HZ, PTE, PER, MI, GER, AD	1.83 [1.00-2.00]	3.00 [2.00-4.83]	0.027
**Group 2 (n=11)**	**Diagnostic accuracy**	**Diagnostic accuracy**	
**Block 1** HZ, PTE, PER	1.00 [0.00-1.00]	1.00 [0.00-1.00]	0.854
**Block 2** MI, GER, AD	1.00 [0.66-1.17]	3.00 [1.33-3.00]	0.006
**Block 3** HZ, PTE, PER, MI, GER, AD	1.66 [1.00-3.00]	4.00 [1.33-4.00]	0.006

Source: Study data. Note: Database: 18 students; p: Wilcoxon test; numerical variables: median [interquartile range]; AD: aortic dissection; GER: gastroesophageal reflux; HZ: herpes zoster; MI: myocardial infarction; n: number of students; PER: pericarditis; PTE: pulmonary thromboembolism; score variation: blocks 1 and 2 (0 to 3); block 3 (0 to 6).

### Case resolution time

Table 3 shows that, in G1, which trained the cases in block 1, there was a reduction in the CRT for all blocks in phase 3. In G2, which trained the cases in block 2, there was a reduction in the CRT for blocks 2 and 3 in phase 3.

**Table 3:** Time spent solving the cases between study phase, by group and block of illnesses

Blocks of illnesses by group	Phase 1	Phase 3	p

Group 1 (n=6)	Time in seconds	Time in seconds	
**Block 1** HZ, PTE, PER	485 [450-583]	318 [284-418]	0.027
**Block 2** MI, GER, AD	558 [400-1,067]	495 [181-646]	0.046
**Block 3** HZ, PTE, PER, MI, GER, AD	1,059 [874-1,744]	812 [466-1,064]	0.028
**Group 2 (n=11)**	**Time in seconds**	**Time in seconds**	
**Block 1** HZ, PTE, PER	501 [485-588]	421 [290-576]	0.328
**Block 2** MI, GER, AD	655 [543-740]	408 [337-569]	0.010
**Block 3** HZ, PTE, PER, MI, GER, AD	1,131 [1,020-1,317]	872 [698-1,062]	0.026

Source: Study data. Database: 17 students (1 case without information); p: Wilcoxon test; numerical variables: median in seconds [interquartile range]; AD: aortic dissection; GER: gastroesophageal reflux; HZ: herpes zoster; MI: myocardial infarction; n: number of students; PER: pericarditis; PTE: pulmonary thromboembolism.

## Discussion

This study evaluated the effect of an instructional approach guided by illness script theory on diagnostic accuracy for cases of chest pain, in medical students. The results confirmed that the methodology improved the students’ diagnostic accuracy and decreased the CRT, suggesting acquisition of mental representation for the illnesses trained, in accordance with illness script theory.^11^ However, transfer of learning to a group of illnesses with the same clinical presentation that were not trained was not confirmed.

Other studies have been guided by illness script theory; however, this is one of the first to imitate the stages of its development. Moghadami et al.^12^ compared the teaching of CR, guided by illness scripts, to the traditional teaching of fourth-year students. The intervention group, after reading a clinical case, was instructed to identify the mental representation of the problem and compare the components of the illness script for 3 differential diagnoses, while the control group attended a lecture on the illnesses in the study and had a small-group discussion. The activity lasted 7 hours, and they found that both groups improved diagnostic accuracy, but the intervention group outperformed the control.

In our study, the participants were less experienced; they were in the beginning of the third year of studies, and they had not started the clinical cycle. It would probably be difficult for these students to identify the mental representation of a problem, as this requires inference abilities and, therefore, greater knowledge about illnesses. Perhaps, in a pre-clinical phase, a methodology that guides cognitive operations for the elaboration of the illness script would be more adequate, and one guided for mental representation of problems could be implemented in subsequent years. These questions may be evaluated in future studies.

In another study guided by illness script theory,^13^ 15 fourth-year and 12 sixth-year medical students participated in a class on illness script theory. Subsequently, the fourth-year students, after reading clinical cases that shared differential diagnoses, informed the common and discriminatory clinical characteristics of each case. Sixth-year students were asked to inform 2 diagnoses, the clinical characteristics of the diagnoses, and the degree of prediction of the clinical characteristics informed. Students received feedback during the activity, which lasted 3 hours.

The results showed that there was an improvement in the ability of sixth-year students to identify new clinical characteristics of the illnesses, with no improvement in diagnostic accuracy and recognition of discriminatory clinical characteristics. Among the fourth-year students, the activity did not demonstrate any benefit.^13^ Unlike our study, the activity was aimed at identifying the clinical characteristics of the diseases, with improved diagnostic accuracy in more advanced students. Perhaps, for less experienced students, a methodology that provides more support, such as the one developed in our study, would have more impact. These considerations may be evaluated in future studies.

Other studies not guided by illness script theory obtained satisfactory results, such as the one carried out by Diemers et al.^14^ who developed a CR course that lasted 10 weeks. In this study, the students explained the pathophysiology aloud, while analyzing 4 cases (2 from the course and 2 from a transfer). Similar to our findings, an improvement in the students’ diagnostic accuracy was observed, with a reduction in CRT, but the learning was not transferred to cases that were not trained. An advantage of the strategy proposed in the present study is the maximum duration of 5 hours, making it feasible in educational environments.

Keemink et al.^15^ investigated the transfer of learning of CR in a course based on clinical cases. After explaining aloud the pathophysiology, predisposing factors, clinical characteristics, workup, and management of 15 diseases (5 from the course), the students analyzed 12 clinical vignettes, 4 with diagnoses trained in the course. Similar to our data, there was an improvement in diagnostic accuracy only for the illnesses that were trained. The debate about the transfer of learning from one context to another is not new.^16^ Transfer of learning is understood to refer to the use of learned abilities to a new situation, which requires the recontextualization of knowledge. This is one of the final stages of learning.^17^

As mentioned, CR occurs through the recognition of illness scripts, which contain the discriminatory clinical characteristics of the diseases.^4^ Studies on analogical transfer suggest that clinical characteristics have both superficial and profound elements. The profound elements relate to the rules that determine a diagnosis, and the superficial elements to the clinical manifestations. For transfer of learning, it is necessary to identify the profound elements; however, the superficial ones are the most noticeable.^17^ In our study, this fact may have prevented the transfer of learning to illnesses that were not trained, because, although they share clinical manifestations, the diagnoses had different clinical characteristics in relation to epidemiology, pathophysiology, and workup. Therefore, the students were not able to arrive at a correct diagnosis, because the specific (in-depth) knowledge of these illnesses was not available. In Medicine, there is no general ability to solve all tasks, given that they have specific contents.^17^

To reproduce the stages of illness script development, this study used suggested educational strategies for teaching CR.^5^ Structured reflection is based on experiential learning that involves perception, description, analysis, and synthesis. It requires that students intentionally search for evidence that supports their learning.^18^ In teaching CR, structured reflection favors the comparison of disease characteristics, contributing to the acquisition of mental representation of them.^19^ Another strategy used was cognitive training, which aimed to exercise some thinking skills,^20 , 21^ such as attention, perception, coding, memory, reasoning, and creativity. Favorable results like this methodology have already been reported for surgical abilities.^20 , 21^ This is one of the first studies to use this methodology in teaching CR, an area that warrants further investigation.

In the end, the students constructed mind maps of the trained diagnoses, which facilitate the visualization of how the information is related, improving memorization of the content.^9^ Kalyanasundaram et al.^22^ demonstrated that mind maps improve information recall 1 week after an instructional activity. Mind maps have been little tested in teaching of CR, but we believe that they may have favored the visualization of the mental representation of illnesses, since their construction considered the components of the illness script.

This study highlights the importance of a structured activity for the development of CR. The results are encouraging, as the literature emphasizes the need for real contact with patients to acquire the illness script.^15^ Our results have shown that, even before beginning clinical activities, students benefit from a CR program, which can serve as a bridge to the beginning of the clinical cycle.

The proposed methodology showed satisfactory results for the teaching of CR. It also allowed exercise of varied diagnoses and manipulation of the components of the illness scripts, which is a relevant fact for the acquisition of an illness script network.^1 , 4^ The activity has an appropriate duration for educational environments, and its incorporation into computing platforms would contribute to greater interaction and feedback. Initiatives with this aim have been developed, such as Clinical Key,^23^ NEJM Healer,^24^ and Paciente 360.^25^

This study has some limitations. This is the only study in this format, with a small number of participants in the final phase. As the study was designed to start before the specialties units, the activities took place concurrently with the final exams in Pediatrics, which precede these units, and this contributed to sample loss. Thus, replication of this study with a greater number of participants would contribute to confirming the results. As the method was tested for the diagnosis of chest pain, it is necessary to evaluate its use for other conditions.

It is not possible to identify the contribution of each strategy used in an isolated manner. It could be argued that the improvement in performance was due to an overall effect of the effort invested in the activity, rather than a specific result attributed to the methodology. Although students dedicated effort to the activity, what matters is that the skill for which they were trained was acquired and that the format may be more attractive in relation to commonly used traditional methods. Work with clinical cases is representative of students’ future practice, and it may favor the development of CR. The feedback received from students was stimulating, as they requested new sessions for other diagnoses.

## Conclusion

The proposed instructional approach improved students’ diagnostic accuracy for chest pain. However, the improvement occurred only for the illnesses trained, and transfer of learning was not observed. The strategy is easy to implement, and it can be considered for the development of CR.
